# A Memoir Containing Observations on the Medicinal Properties of Oxygen

**Published:** 1799-08

**Authors:** 

**Affiliations:** sen. Surgeon of the First Class, to the Armies of the Republic, First Surgeon to the Military Hospital, and Member of the Society of Medicine of Brussells, and of the Lyceum of Arts at Paris


[*In laying the following Memoir before our Readers, we have not the ſmalleſt intention of rendering them any ſignal information, but they will thereby have an opportunity of comparing the ſtate of Medical Surgery in France, with that of England.*]

---

### *A Memoir containing Obſervations on the Medicinal Properties of Oxygen.*

By Cit. FOURNIER, sen. Surgeon of the First Class, to the Armies of the Republic, First Surgeon to the Military Hospital, and Member of the Society of Medicine of Brussells, and of the Lyceum of Arts at Paris.

(*Read to the Medical Society of Paris, the 17th Thermidor, 6th Year.*)

OCCIDIT QUI NON SERVAT.

CITIZEN ALYON has rendered an important ſervice to the healing art, in eſtabliſhing, by authentic experiments, the medicinal properties of oxygen.

The

The author has, with great ingenuity, explained how this principle of animal exiftence may be ufefully employed in different difeafes, in the treatment of which, the means of cure formerly employed were moftly difagreeable to the patient, fometimes uncertain, and liable to produce diftreffing confequences.

He has explained to thofe who are not entire ftrangers to chemical proceffes, that mercury probably acts in venereal affections only by the oxygenation which it acquires in its different preparations, previoufly to its being adminiftered.

We already know that, under whatever form mercury is employed, it is indebted for its anti-fyphilitic virtue, to the oxygen with which it is combined.

But what we are ftill ignorant of is, how the oxygen alone can counteract the venereal virus, and other difeafes, for the cure of which the fpecific hitherto ufed was mercury.

Struck with this remarkable fact, announced by Alyon and Girtanner, I wifhed to be convinced of its reality by a feries of experiments. Certain of the fafety of this medicine, I did not hefitate to introduce it in my practice; and its fuccefs has fully anfwered my expectations.

Among feveral obfervations I have felected, I fhall lay before the Society a few which will be found interefting.

Citizen L. about 40 years of age, confulted me for relief in pains of the bones, which afflicted him extremely, and prevented him from fleeping. As this man had lived with a woman who was fo fhockingly affected with the venereal virus, that it had caufed her death, I did not doubt that the pains of my patient were the fymptoms of this cruel difeafe. I twice ordered refrefhing diluents, and the domeftic baths, as preparatives for the mercurial treatment to which I had refolved to fubmit him. After he had bathed fix times, there appeared between the prepuce and the gland two large and deep chancres. The antiphlogiftic regimen to the ufe of which the patient had been accuftomed, was probably the reafon why the chancres were not attended with great inflammation; but they were neverthelefs extremely painful, and gradually increafed in fize. I prefcribed purgatives, and propofed mercurial treatment, to which he obftinately refufed to fubmit, being apprehenfive of experiencing the fate of the woman before alluded to, whofe life mercury could not fave. In another inftance, I would have adminiftered the anti-fyphilitic robe of Laffecteur, from which I have witneffed aftonifhing effects, in cafes where mercury had proved abortive. But excited cited by an ardent deſire of trying whether oxygen was really worthy of the eſtimation in which it was held by Alyon, &c., I took this opportunity of preſcribing it. I daily gave the patient a drachm of pure nitric acid in two pounds of water, and he had only taken this drink eight days, when the chancres, which were broke and had produced an ichorous and abundant ſuppuration, cleanſed, became red, and began to cicatrize.

On the twelfth day the chancres diſappeared; the patient was then purged. The pains of the bones ſtill continued, and he was as much troubled with them now as formerly. I increaſed the doſe of the acid to a drachm and a half a day: ſoon after the pains began to diminiſh, and the patient was able to move his right arm with eaſe, which he could not do before. His ſleep gradually returned, and by the thirtieth day the cure was completed. The patient took a cathartic, and continued the daily uſe of the nitric lemonade, which had been increaſed in ſtrength to two drachms in three pounds of water, ſince the 25th day. He left off drinking it on the thirty-eighth day, when an abundant ſalivation took place, which was ſubdued by a purgative, and twice warm bathing. The patient was then perfectly cured, and has ſince continued in good health.

Citizen G. twenty-four years of age, of a humid and ſanguine conſtitution, after an impure connection, was infected with a virulent gonorrhœa. This was the firſt accident of that nature he had met with. The local inflammation was conſiderable, and conſequently accompanied with acute pain. I adviſed him to drink plentifully of dog-graſs and marſh-mallow roots; I alſo bled him freely, and ordered the warm bath. As the diſcharge was thick, and mixed with blood, and his urine in ſmall quantity, and voided with difficulty, I preſcribed pills compoſed of Venice ſoap, camphor, nitre, and rhubarb, and alſo an anodyne emulſion every evening.

Theſe remedies produced the effect I expected; the pains abated, the diſcharge became abundant, and acquired a proper colour and conſiſtence.

Some time elapſed before the patient called on me again; and either from his inattention to a proper regimen, or from the diſeaſe having acquired a more virulent character, he was afflicted with a conſiderable inflammation in his eyes, accompanied with a great degree of fever. The violence of this ſymptom, and the ſudden diſappearance of the gonorrhœal diſcharge, impreſſed me with an idea, that his caſe was venereal. I adopted baths, bleeding, collyria of zinc; plaſters made of bruiſed lintſeed, the whites of eggs, and vitriolated zinc, were applied to the parts affected, as alſo a decoction of dog-graſs, acidulated with tartarit of potaſs and antimony, and one ounce of of ſulphat of magneſia to each pot. I alſo applied eight leeches to the lower eye-lids, which were much ſwelled.

All theſe means appeared to have a ſalutary effect; but I had ſcarcely began to congratulate myſelf, when the virus, which before was fixed in the eyes, was ſuddenly tranſlated to the bend of the right knee, which became ſwelled and painful. I oppoſed this new Proteus by vegetable diet, once bleeding, baths, purgatives, and emollient and reſolvent cataplaſms. The ſwelling and pain now began to decreaſe, when a violent thunder-ſtorm, which was approaching, excited the curioſity of the patient, who placed himſelf at a window, to obſerve the flaſhing of the lightning.

This imprudence had nearly coſt him his life, for ſuddenly his ophthalmia re-appeared with greater violence than before. I had recourſe to the ſame general means which I had formerly employed with ſucceſs, and the inflammation again ceaſed, but the patient became almoſt blind. The cornea of each eye was dull, and the iris contracted very little, when oppoſed to candle-light. I ordered a large veſicatory to be applied to the nape of his neck, with a view to prevent the amauroſis, as well as to cauſe a diſcharge of the morbific matter, which again threatened to return to the knee.

I now preſcribed oxygen (about this time the oxygen had juſt taken effect on the patient mentioned in the preceding obſervation). I gave him four ſcruples in three pounds of water daily. The diſcharge from the urethra ſoon returned, but more moderately than formerly, and the ſuppuration of the veſicatory continued.

The eighth day after the application of the oxygen, a purgative was given. No other ſymptoms intervened on the knee, and the ophthalmia aſſumed a convaleſcent appearance. The cornea of both eyes ſtill continued rather dull, but the action of the iris was reſtored. On the twentieth day the ophthalmia was entirely cured, but the eye was painfully affected by light. The running from the urethra, which now reſembled ſemen, announced the deſtruction of the virus.

At this ſtage of the diſeaſe, the patient was ordered to take another cathartic, which was repeated on the thirtieth day, when I conceived him to be cured. He continued the uſe of the nitric lemonade till the fortieth day, when it was diſcontinued, as was alſo the veſicatory. Several laxatives completed the cure, and for a month the patient has enjoyed a perfect ſtate of health.

At the moment I now write, I attend an officer, who has for ſeveral years been afflicted with venerea ltetters on his upper lip, noſe, and forehead; theſe

 tetters tetters appeared in the form of difgufting puftules, and were exceedingly troublefome. Either in confequence of his natural obftinacy, or by the hardfhips which attend a foldier's life, the patient had unfuccefsfully tried various mercurial and other preparations. He at length applied to me; I advifed him to drink the nitric lemonade, made with a drachm of acid to two pounds of water, and directed him to wafh the tetters every evening with the oxygenated pomatum, defcribed by Alyon. By this treatment, which was fcarcely continued twelve days, two purgatives, and feveral bathings, the eruptions entirely difappeared, and the patient, fuppofing himfelf to be cured, had difcontinued the medicines for the laft ten days; but I advifed him to recommence them, apprehenfive that the cure was not yet perfect. I may venture to affert, that he will be completely cured, provided he follows my directions for another fortnight—the time I believe to be indifpenfable to eradicate fo inveterate a virus, which had fo long refifted the beft combination of remedies.

I have already obferved, in feveral venereal cafes, the good effects of oxygen, exhibited as an auxiliary to mercury. Several diftinguifhed practitioners of this city, have made fuccefsful trials with this remedy; and one of them informed me of the following interefting fact, which is decifively in favour of oxygen.

A young man twenty-two years of age, was attacked from his infancy with a fcorbutic humour *(humeur dartreufe)*, over his whole body, his hands and face excepted; and feveral phyficians had unfuccefsfully prefcribed venefection. The practitioner who communicated this obfervation to me, after having prepared his patient by general remedies, employed the nitric lemonade, and directed him to rub every evening, a certain part of his body with the oxygenated ointment; after this treatment had been purfued forty days, affifted by purgatives and bathing, the young man was completely cured. Thus has oxygen produced effects in this fhort lapfe of time, which the ufual medicines could not accomplifh during twenty years.

The following is a fact of a different nature, which is not lefs interefting:

A very delicate child, tormented with a colic, which produced frequent convulfions, had attained the age of two months, when a fevere catarrhal affection deprived its mother of her milk. The infant was fed with fpoonmeat, and a variety of panadas which were fubftituted for its natural aliment. At the end of fix days it was feized with hoarfenefs, which, on account of the frequency, abundance, and crudity of its ftools, was deemed alarming. The next day a wet-nurfe was fent for, in hopes that human milk would be the moft efficacious of all remedies. The diarrhœa continued, and the child child did not appear to fuffer pain, but feemed more exhaufted at every ftool. All the ufual remedies were adminiftered without effect. Let it fuffice to obferve, that the affiftance of art was not neglected, when my efteemed friend, Profeffor KOK, aided me with his advice in the treatment of this infant. On the fifth day all our hopes were at an end, and at five o'clock in the evening, the patient was at the laft extremity. It had fuch a copious ftool that I thought it would have expired through exhauftion. All the fymptoms of death were manifeft in its countenance; it could only juft breathe; and it paffed the night without being able to fuck or fwallow any thing; it no longer voided urine. The next morning, Kok and myfelf conceived the child was at the point of death; the pulfe was irregular, flow, debilitated, and almoft imperceptible, the belly inflated, (*météorifé*) the cornea dim, the eye filled with purulent matter, the face pale and hippro-cratic, the extremities chilly, and a fetid and gelatinous matter iffued from the mouth and noftrils. Kok undertook the melancholy tafk of announcing to an affectionate mother, that we had no hopes left of faving her infant. She propofed to re-animate the child, who could no longer fwallow any thing, by anointing its lips with excellent Mufcadel wine; we confented to this: and at that moment an idea ftruck me, at which I have fince had much reafon to rejoice.

I haftened to perufe the work of Alyon, on the properties of oxygen; I had read in the collection of the Medical Society of Paris, a memoir by their fecretary, SEDILLOT, junior, in which he gives an account of cafes of rheumatifm, cured by frictions with acetic ether on the parts affected; and, as I am often afflicted with this tormenting difeafe, I had provided myfelf with fome of this liquid from Paris (the application of which I had fuccefs-fully prefcribed to a lady contracted by chronic rheumatifm). I recollected that acetic ether is one of the pharmaceutical preparations, in which oxygen is in a free ftate, and confequently, as this ether is of a very fedative and not ftupifying quality, it appeared to me to be a proper application for the child, which had juft experienced a paroxyfm which lafted fix minutes; during which, the fufpenfion of the pulfe and refpiration, induced us to fuppofe that it was dead. The acetic ether could only reftore the almoft extinguifhed irritability, and ftimulate the vital principle; and as we apprehended that every minute would be its laft, I thought there could be no rifk in adminifter-ing a remedy, which, according to the lateft analyfis, could not prove hurtful. I put a drachm in the fame quantity of Mufcadel wine, and frequently applied this mixture to its mouth and throat with a feather. After the expiration of feveral hours, I perceived the pulfe increafing, and that the child was not infenfible of the application, which excited irritability, when introduced into its its mouth, and that it was ſoon enabled to ſwallow a few drops of this mixture. I redoubled my aſſiduity till ſix o'clock in the evening, during which time the infant ſwallowed two drachms of the ether, when a happy change took place. It is worthy of remark, that in the ſpace of ſix days, the effects of the diſeaſe had been ſo violent, that the child had fallen into a complete maraſmus. I have already obſerved that it ſuffered little, and during twenty-five hours of the paroxyſm, it had no convulſions, which may be attributed to the colics which had preceded. In the courſe of the twenty-five hours it uttered ſome feeble cries, though not often; it neither had ſtools nor made urine; but at ſix o'clock at night it began to cry oftener, and louder; and it had a ſtool more looſe than the former; from theſe appearances, I inferred that the remedy had taken effect, and began to entertain hope. I put one ounce of ſyrup of poppies and a drachm of the extract of Peruvian bark into four ounces of ſtrong beef broth, and adminiſtered this mixture in a glyſter; it immediately ejected one half, and a ſecond injection was given, which it retained twelve hours; the inſtant the glyſter was given, an abundant diſcharge of urine took place. I immediately ordered the child to be put to bed, and gave it ten drops of the *acetic ether* in a teacup full of ſtrong beef-tea. My joy and ſurpriſe cannot eaſily be imagined, when I ſaw it ſwallow this mixture! I repeated the drink ſeveral times, and its pulſe increaſed gradually; its extremities acquired a gentle degree of heat, which rapidly increaſed, and at ten o'clock at night, a violent fever was manifeſt by its pulſe, which beat 150 times in a minute; and at this period, a profuſe perſpiration took place over the whole of its body, particularly the head. I ſuſpended the adminiſtration of acetic ether, which having produced the happy change I have juſt deſcribed, could no longer be of utility; and confined myſelf to the moiſtening of the throat with beef tea. At two o'clock in the morning the fever abated, the perſpiration ceaſed, and this criſis was ſucceeded by ſleep. The child did not awake till ſix o'clock, when the glyſter of the preceding evening operated, which was replaced by another of the ſame compoſition. At eight o'clock the wet-nurſe having preſented it the breaſt, it took it with avidity, but it could ſuck but little, in conſequence of its weak ſtate.

During the two following days, the child had an almoſt continual ſleep, which was interrupted only by the ſtools, amounting to twelve or thirteen in number. As ſoon as it had had one, I injected the glyſter before mentioned, to which I added the yolk of an egg, with a view of ſtrengthening and nouriſhing the patient, and of giving tone to the inteſtines. The breaſt and a cup of the broth, in which I put ſome ſyrup of bark, were taken alternately, from which its little diſordered ſtomach experienced good effects.

For

For the laſt two days, the child has appeared freſh coloured and eaſy, and as if it had juſt awoke from a long ſleep.

The matter which filled its eyes, and which had dried, cauſed much pain before it could be removed; notwithſtanding which, thoſe organs, to my great aſtoniſhment, have remained uninjured.

This child is now four months old, it poſſeſſes the ſtrength and intellect of one of ſix months, and enjoys perfect health. It has never ſince ſuffered the ſmalleſt indiſpoſition, and is lively and thriving.

Is the preſervation of this child to be attributed to Nature alone, or to the oxygen? All that I have above related, induces me to attribute it principally to the latter cauſe, as I have ſeen it operate in a ſenſible manner; and to it I decidedly attribute this almoſt miraculous reſtoration.

Such are the obſervations which I reſpectfully ſubmit to the Medical Society; on this occaſion, I have no other ambition than to ſecond the zeal which it conſtantly employs, for the progreſs of the healing art, and the good of mankind. My work does not diſplay the ornaments of ſtyle and elegance of erudition, it only has the merit of preciſion, and the moſt ſcrupulous adherence to truth. Enthuſiaſm ought not to form a part in the character of the phyſician, becauſe it is ſeldom conſiſtent with truth. It is with a deſign of rendering homage to the latter, that I ſhall here give my opinion on the gaſtric juice, which has been ſo much extolled as the means of introducing medicinal ſubſtances into the human body. In conſequence of the invitation given to practitioners by the Society, in its "*Recueil Periodique*," to eſtabliſh, by experiments, the efficacy of the means propoſed by Brera, I have attempted to admininiſter different remedies by this proceſs. Opium produced no effect. I have given the *(diagredium)* ſcammony as well as the reſin of jalap (of which a doſe of 15 grains was a purge) a drachm for each doſe, unleſs the patient was much affected; and I applied frictions, ſometimes to the ſtomach and ſometimes to the abdomen, without ſucceſs. The antimonial tartar had the ſame effect, taken at about 6 grains for a doſe. The corroſive ſublimate, adminiſtered in frictions on the extremities of three venereal patients, one of whom had a bubo and chancres at the penis, and the other two had chancres only, had the deſired effect.

Thirty grains were ſufficient for each time of friction. But, is it by means of the gaſtric juice that this remedy ſuceeded? Would it not have had the ſame effect if it had been introduced into the abſorbent veſſels, incorporated with hog's lard or any other proper vehicle? This is what I maintain in the affirmative, as the reſult of my experiments.

At

At the time I made these trials at the Military Hospital at Brussels, where I was surgeon in chief, Dr. Duval, my colleague at the same hospital, made similar trials among his febrile patients, without better success. He observed that the bark left, during some minutes, a lively colour, accompanied with heat, on the part of the stomach or abdomen to which it had been applied. He seemed to think that squills, given in a triple dose, had in one particular instance increased the quantity of urine.

After having made experiments on the gastric juice of several herbivorous animals, and the human saliva, with no better success, we have rejected that doctrine, the supporters of which are, without doubt, under illusion.

